# Species assemblages of aquatic and semi-aquatic true bugs (Hemiptera, Heteroptera) of anthropogenic ponds of the green zone in Bangkok, Thailand

**DOI:** 10.3897/BDJ.13.e154135

**Published:** 2025-05-29

**Authors:** Natthapont Janthachid, Boonsatien Boonsoong, Kulchon Diloknorranart, Akekawat Vitheepradit

**Affiliations:** 1 Department of Entomology, Kasetsart University, Bangkok, Thailand Department of Entomology, Kasetsart University Bangkok Thailand; 2 Department of Zoology, Faculty of Science, Kasetsart University, Bangkok, Thailand Department of Zoology, Faculty of Science, Kasetsart University Bangkok Thailand; 3 Research and Lifelong Learning Center for Urban and Environmental Entomology, Kasetsart University, Bangkok, Thailand Research and Lifelong Learning Center for Urban and Environmental Entomology, Kasetsart University Bangkok Thailand

**Keywords:** Heteroptera, diversity, aquatic insects, urban

## Abstract

Aquatic and semi-aquatic true bugs (Hemiptera, Heteroptera) are highly abundant in both lentic and lotic systems in Thailand. More than 230 species representing 12 families of these insects have been reported from freshwater habitats in Thailand. The Thai fauna has been studied intensively in the last several decades, but no research focuses on the species assemblages of aquatic and semi-aquatic true bugs in urban areas. Two important discoveries were found in this study: the species assemblages of aquatic and semi-aquatic true bugs of ponds in the green zones of Bangkok and their association with margin type (natural vs. artificial).

The complete taxonomic list of aquatic and semi-aquatic true bugs from 28 ponds in the green zones is presented. Twenty species representing 16 genera, 12 families and two infraorders are reported in this study. More specially, nine species, seven genera and four families of Gerromorpha and eleven species, eight genera and seven families of Nepomorpha were collected. Although the number species of this research accounts for only 9% of the Thai fauna, the study area represented less than 0.01% of total land area in Thailand. Overall, Cluster Analysis and Principal Component Analysis showed species compositional dissimilarities between ponds with natural margins and ponds with artificial margins. In general, species richness, especially members of Nepomorpha in ponds with natural margins, is higher than those in ponds with artificial margins. This finding is valuable for aquatic system management to promote a higher diversity of aquatic insects in the green zones of urban areas in Thailand.

## Introduction

Bangkok, the capital of Thailand, is in the heart of the country at N 13^o^.45^’^ E 100^o^.28’ with an elevation between 1–2 m above sea level (Sinsakul 2000). Bangkok covers approximately 1,600 km^2^ and with a population of more than five million people, is one of the most populated cities in Southeast Asia (Douglass 2004). Based on the most recent land-use survey, green areas occupy less than 50 km^2^ or 3.40% of Bangkok (Bangkok Metropolitan Administration, Department of City Planning and Development 2023). The majority of land use is residential and business and a small amount of agriculture (Bangkok Metropolitan Administration, Department of City Planning and Development 2023). To maximise the green zone of Bangkok, the Bangkok Metropolitan Administration (BMA) established forty recreational parks around Bangkok (Bangkok Metropolitan Administration, Environment Department 2023). Although these recreational parks vary in size and landscape design, they are strictly managed and operated by the BMA. One of the fundamental objectives of these recreation parks is to serve as natural habitats for wildlife in Bangkok’s urbanised ecosystem. Lately, numerous urban ecological projects and activities have been hosted in these recreation parks to increase awareness of the value of nature and importance of biodiversity in Bangkok (Nguyen and Chidthaisong 2023).

During the last decade, various studies on diversity in green zones within Bangkok have been conducted, especially on birds (Sananunsakul et al. 2017, Chaiyarat et al. 2018, Chankhao et al. 2023, Chankhao et al. 2024, Uwichain and Danaisawadi 2024). Few studies have focused on the diversity of insects (Stewart et al. 2018, Jaturas et al. 2020). No studies of aquatic insect diversity in Bangkok have been conducted. Aquatic and semi-aquatic true bugs belong to two infraorders within the order Heteroptera: Gerromorpha that live on the water surface and Nepomorpha that live below the water amongst aquatic vegetation in the littoral zone. In Thailand, much more research has been conducted on aquatic and semi-aquatic true bug diversity in forested areas than in urban areas (Vitheepradit and Sites 2007, Sites and Vitheepradit 2011, Nakthong et al. 2014, Raruanysong et al. 2014, Attawanno and Vitheepradit 2022, Sritipsak and Vitheepradit 2023, Vitheepradit et al. 2024). Additionally, few studies have focused on the fauna of aquatic and semi-aquatic true bugs within lentic systems in Thailand (Vitheepradit 2008, Sites and Vitheepradit 2011). Therefore, urban freshwater ecosystems, such as canals, ponds and reservoirs have received little attention despite their potential to support diverse aquatic insect communities (Ferzoco et al. 2023).

Aquatic true bugs have developed various physiological and behavioural adaptations that allow them to thrive in lentic systems (Paul and Datta 2022, Bonada and Bogan 2024). Many species possess specialised respiratory structures, such as plastron respiration, which enables them to extract oxygen directly from the water, reducing their reliance on surfacing for air (Chen et al. 2021, Kumar et al. 2023). Additionally, giant water bugs (Belostomatidae) use air-breathing siphons or trapped air bubbles to sustain underwater respiration for extended periods (Lee 2024). Additionally, their ability to tolerate environmental conditions, including changes in temperature, dissolved oxygen and water chemistry, makes them well−suited for lentic habitats, which often experience seasonal variations in water quality (Dettner 2019, Andersen and Weir 2004). In urban environments, lentic habitats such as reservoirs, ponds and artificial wetlands are crucial for sustaining aquatic biodiversity (Savić et al. 2021, Hill et al. 2023). However, these habitats face significant ecological pressures, including pollution, habitat fragmentation and invasive species introduction. Despite these challenges, aquatic true bugs continue to remain in urban lentic systems, demonstrating their adaptability to withstand anthropogenic disturbances. The relationships between aquatic true bugs and the presence of natural surroundings are not clearly known, but several research studies indicated aquatic true bug richness in ponds was with natural surroundings comparing to those surrounded by man-made surroundings (Nakanishi et al. 2013, Keinath et al. 2023). Given the rapid urbanisation of Bangkok, understanding the diversity and ecological functions of aquatic insects in the city freshwater systems is crucial for effective urban conservation planning and water resource management. Understanding the species composition and ecological roles of these insects in lentic systems is essential, as these habitats are common in urban areas and may serve as critical refuges for aquatic biodiversity. The following research provides the first species-level survey of aquatic and semi-aquatic true bugs (Hemiptera, Heteroptera) from lentic habitats in the green zone of Bangkok and their association with marginal types.

## Materials and methods

### Study area

**Figure 1.**
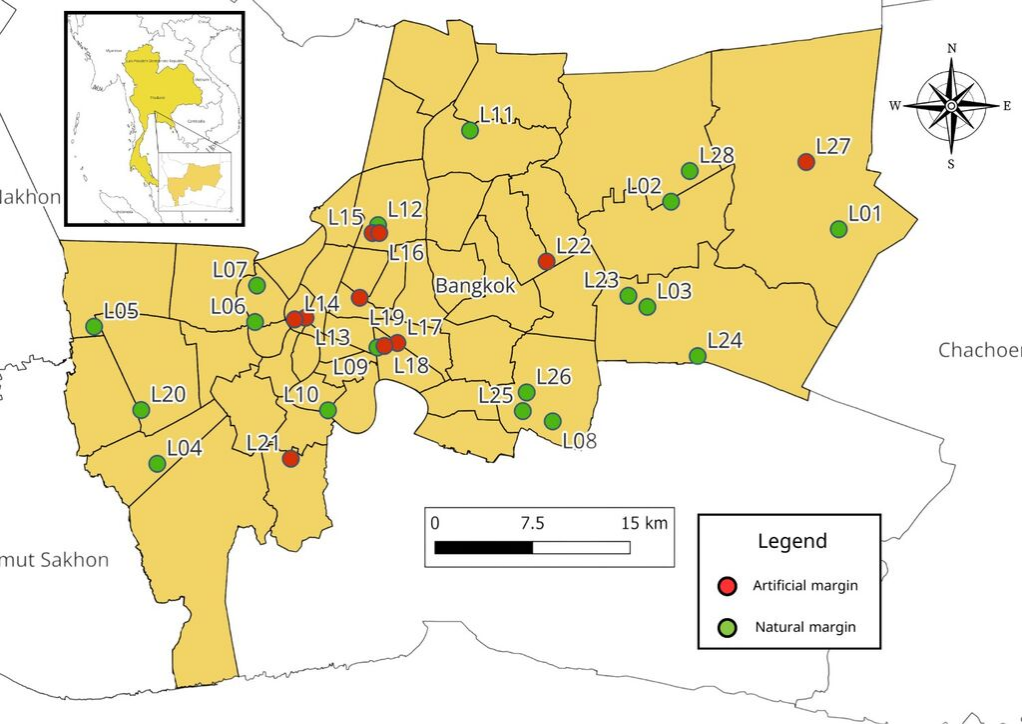
Sampling sites within Bangkok. Pond coding is presented in Table 1.

**Figure 2.**
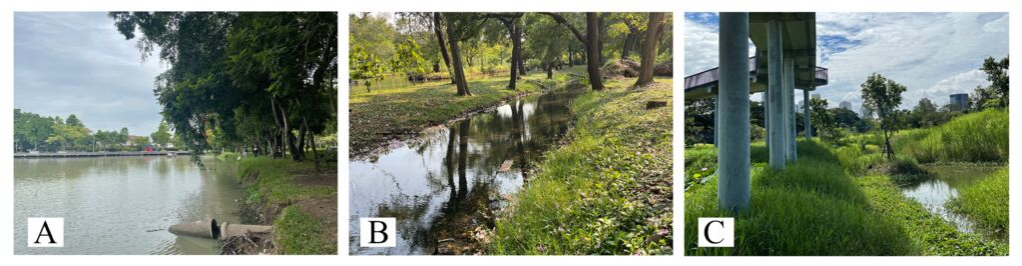
Ponds with natural margins: **A** Her Majesty the Queen's 60th Birthday Park (L23); **B** Wachirabenchatat Park (L12); **C** Benchakitti Forest Park (L9).

**Figure 3.**
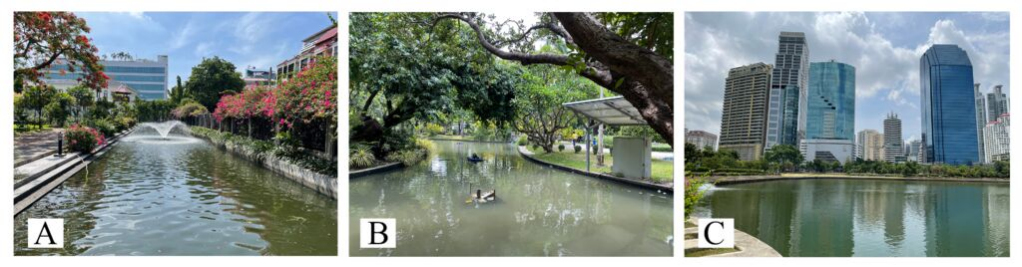
Ponds with artificial margins: **A** Rommaninat Park (L13); **B** Saranrom Park (L14); **C** Benchakiti Park (L18).

Aquatic and semi-aquatic true bugs were collected from 28 ponds in 22 districts within the Bangkok green zone (Fig. 1, Table 1). Each pond was sampled twice, first during 2021 and again during 2022 (56 samples total). All ponds were similar in size and managed by the BMA. Although these ponds were heavily anthropogenically influenced lentic habitats, they could be further categorised into two groups: ponds with natural margins and ponds with artificial margins. Pond margins were considered “natural” when the margin consisted of soil with rooted vegetation at the water’s edge and/or continuing into the water (Fig. 2). Margins were considered “artificial” when the margin consisted of concrete with little to no rooted vegetation (Fig. 3).

Eighteen ponds with natural margins and 10 ponds with artificial margins were surveyed in this study.

#### Sampling and Identification

Ponds were quantitatively and qualitatively sampled for their aquatic and semi-aquatic true bug fauna from two mesohabitats (i.e. margins and water surface) by following the collecting protocol in Vitheepradit (2008). For quantitative sampling, three samples of each mesohabitat were collected from a random location along the margin at each pond. Aquatic samples were collected using an aquatic D-net, although the specific sampling method differed between mesohabitats. For margins, samples were taken by sweeping the net beneath the water surface, against and parallel to the margin, back and forth three times to collect littoral zone insects (e.g. Nepidae, Naucoridae). For water surface samples, an aquatic D-net was swept at the water surface parallel to the water margin three times to collect surface-dwelling insects (e.g. Gerridae, Veliidae).

For qualitative collection, one sample of each mesohabitat was collected from each pond. The D-net was swept over the water surface to collect neuston insects or swept through the margins of each pond to collect marginal insects. In each of these sampling regimes, when the D-net was up to 1/3 full, the contents were transferred to a white pan. In each mesohabitat, sampling continued until no recognisably new morphospecies were collected in two consecutive samples.

All samples were sorted in the field using soft forceps to remove specimens, which were placed into containers with 80% ethyl alcohol. Samples from each mesohabitat were labelled and put in separate containers. At each pond, a Global Positioning System (GPS) was used to record latitude, longitude and elevation (WGS84 datum). Specimens were identified to the lowest level possible, generally species, using a stereomicroscope and appropriate taxonomic keys, such as: Vitheepradit et al. (2003), Yule and Yong (2004) and Cook et al. (2020). Specimens were photographed using a Leica EZ4W stereomicroscope coupled with the LAS EZ programme. Images were then prepared with Photoshop CS5 (Adobe Systems Inc., San Jose, CA, USA). Specimens were deposited in the Entomology Museum, the Department of Entomology, Kasetsart University, Bangkok, Thailand (EMKU).

### Analysis

Both year’s data were pooled for all samples. Taxonomic richness and abundance were tested for a normal distribution using the Shapiro–Wilk test by SPSS version 28 (IBM Corp. 2021). Student’s t-test was performed to test whether there was a difference in abundance of Gerromorpha, Nepomorpha or All taxa by SPSS version 28 (IBM Corp. 2021). Principle Component Analysis (PCA) was used to determine species richness and abundance patterns of aquatic and semi-aquatic Heteroptera between natural and artificial margins. In addition, Cluster Analysis was used to assess the compositional similarity in richness/abundance and between natural and artificial margins. These analyses were performed using PC-ORD software 5.0 (McCune and Mefford. 2016).

## Data resources

Specimens were collected with the permission by the Institutional Animal Care and Use Committee, Faculty of Agriculture, Kasetsart University (Thailand) under project number ACKU67-AGR-040 and ACKU68-AGR-005.

## Results

### Taxonomic results

**Figure 4.**
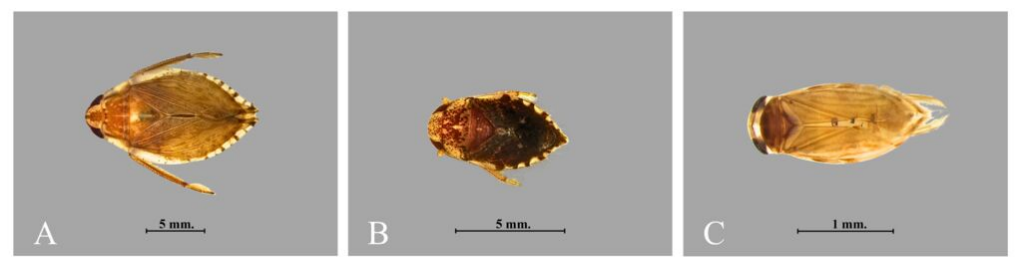
Representatives of Nepomorpha collected during this study: **A** *Diplonychusrusticus*; **B** *Thurselinusscutellaris*; **C** *Micronectascutellaris*.

**Figure 5.**
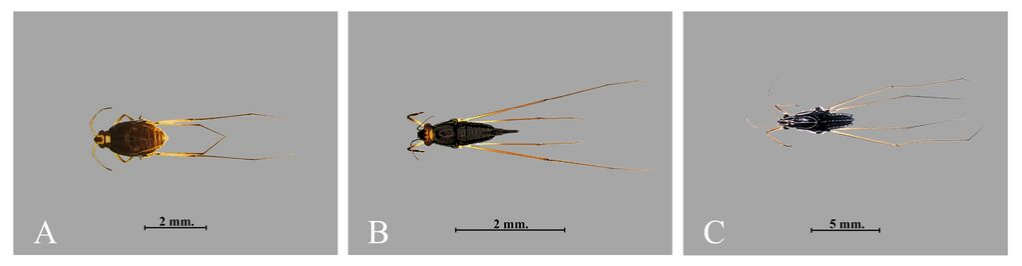
Representatives of Gerromorpha collected during this study: **A** *Naboandelussignatus*; **B** *Rhagadotarsuskraepelini*; **C** *Limnogonusfossarum*.

**Figure 6.**
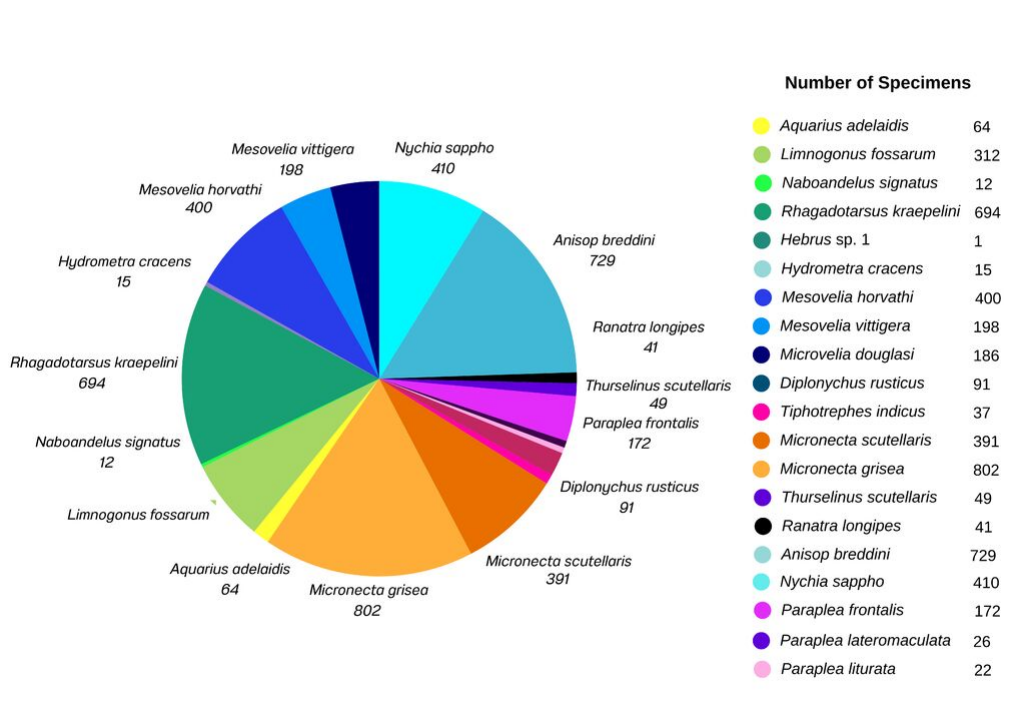
A pie chart representing abundance of each species collected in this study. Number indicates numbers of specimens of each species.

A total of 4,652 specimens, representing 20 species, 16 genera, 12 families and two infraorders of Heteroptera were collected in this study (Figs 4, 5, 6, Table 2). All specimens were identified to the level of species, except a single female specimen which could only be identified to genus. The most species rich family was Gerridae, with four species, followed by Pleidae with three species, Mesoveliidae, Micronectidae and Notonectidae with two species and Belostomatidae, Hebridae, Helotrephidae, Hydrometridae, Naucoridae, Nepidae and Veliidae with a single species each. Overall, 19 species were recorded from ponds with natural margins, whereas 14 species were collected from ponds with artificial margins. *Naboandelussignatus* (Gerridae) was the only taxon that was absent from ponds with natural margins. All species in Nepomorpha were commonly found in every pond with natural margins, except for *Paraplealateromaculata* and *Paraplealiturata* which were collected from only two ponds (L01 and L02). Eight of 11 Nepomorpha species were collected from ponds with artificial margins (Table 1). Specifically, *Ranatralongipes*, *P.lateromaculata* and *P.liturata* were absent from all ponds with artificial margins, but *Micronectascutellaris* and *Anisopsbreddini* were each only collected from a single pond with artificial margins (Table 2).

### Analytical results

Student's t-test based on abundance of Nepomorpha and all taxa were significantly different in abundance between natural margin pond and artificial margin pond with *p* = 0.010 and *p* = 0.009, respectively. On the other hand, there was no significant difference in abundance of Gerromorpha (*p* = 0.392) (Table 3).

**Figure 7.**
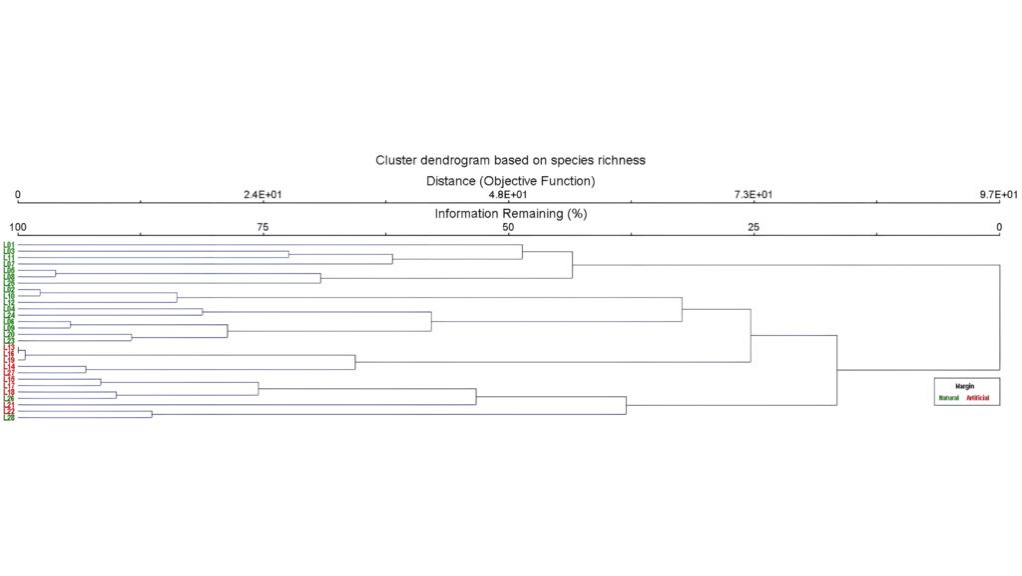
Cluster dendrogram, based on species richness of aquatic and semi-aquatic true bugs from natural (green) and artificially-margined ponds (red).

**Figure 8.**
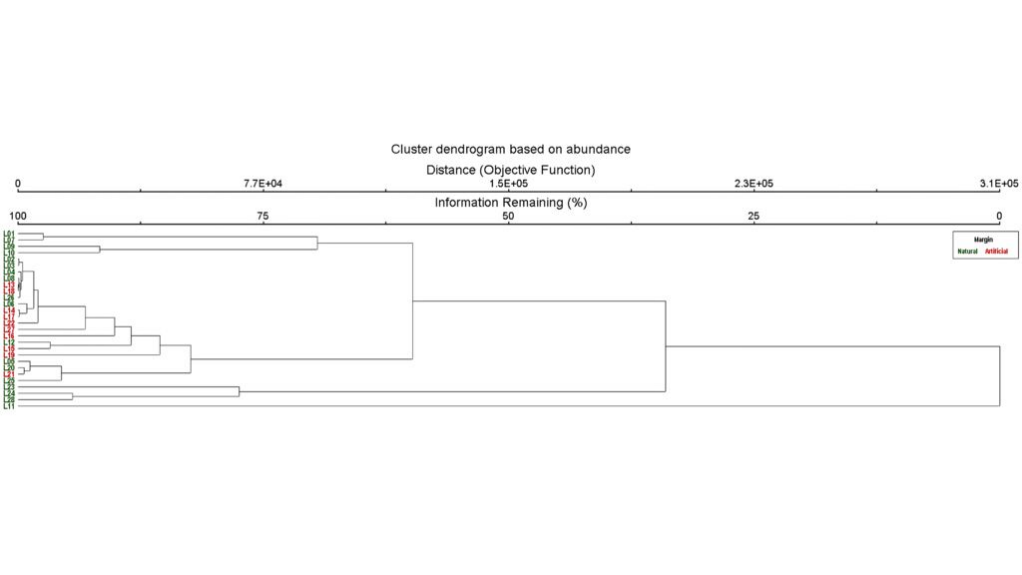
Cluster dendrogram, based on abundance of aquatic and semi-aquatic true bugs from natural (green) and artificially-margined ponds (red).

Cluster analysis, based on species richness, resulted in two main branches showing distinct groups between ponds with natural margins and ponds with artificial margins (Fig. 7). Only two ponds with natural margins (L26 and L28) were grouped with artificially-margined ponds (Fig. 7). Cluster analysis, based on species abundance, showed similar, but less clear-cut results (Fig. 8). Four main branches were created, three of which correctly clustered naturally-margined ponds, the fourth combined all artificially-margined ponds with a few naturally-margined ponds (Fig. 8).

**Figure 9.**
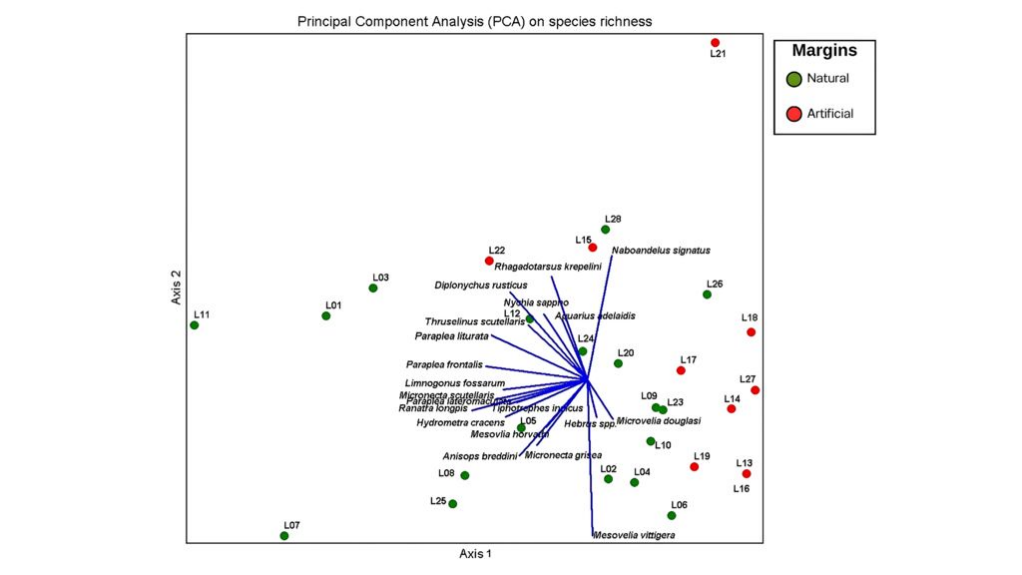
PCA using species richness of aquatic and semi-aquatic true bugs from natural margin ponds (green) and artificial margin ponds (red).

**Figure 10.**
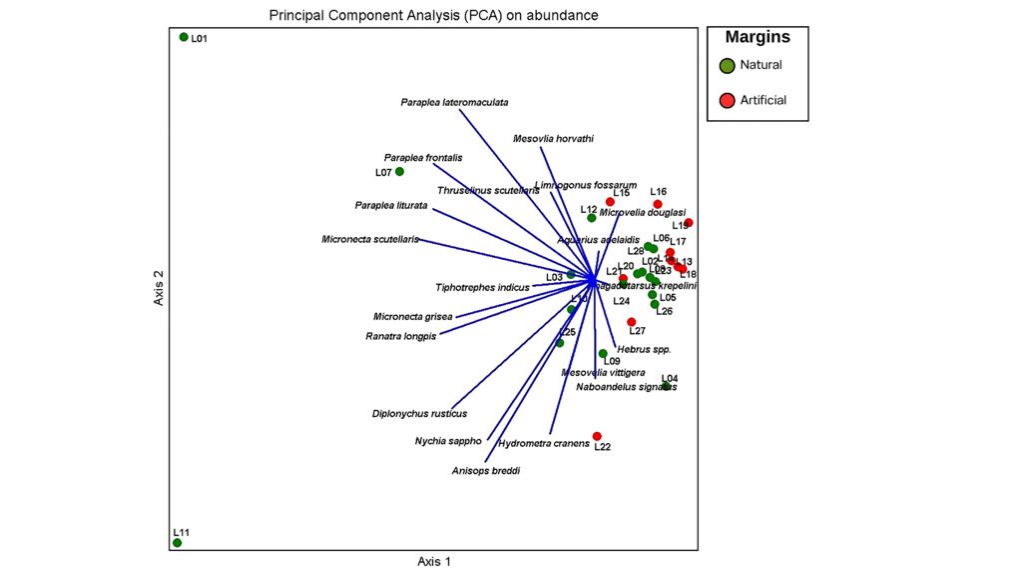
PCA using abundance of aquatic and semi-aquatic true bugs from naturally- (green) and artificially-margined ponds (red).

Similar to cluster analysis, principal components analysis, based on richness, was better able to distinguish between the natural and artificially-margined ponds (Fig. 9) than a PCA based on abundance (Fig. 10). Ten species of Nepomorpha and eight species of Gerromorpha were associated with the group of naturally-margined ponds, whereas a single species of Gerromorpha was associated with artificially-margined ponds. Using abundance data, half of the naturally-margined ponds were closely aligned to the group of artificially-margined ponds (Fig. 10). In addition, 10 species of Nepomorpha were associated with the group of naturally-margined ponds, whereas four species of Gerromorpha were associated with the group of artificially-margined ponds.

## Discussion

Several faunistic surveys of aquatic and semi-aquatic true bugs of ponds in Thailand have been conducted. Vitheepradit (2008) examined the Gerromorpha species assemblages of 88 ponds adjacent to the northern, western and southern mountain ranges in Thailand. The results revealed 24 species representing 11 genera and four families of Gerromorpha from these lowland ponds. Secondly, ten unaffected ponds in the south of Thailand and 12 ponds inundated by a Tsunami were sampled four times during 2005–2006 to determine the species composition of aquatic insects, including aquatic and semi-aquatic true bugs (Sites and Vitheepradit 2011). They collected 22 species representing 10 genera and four families of Gerromorpha and 28 species representing 13 genera and eight families of Nepomorpha from the unaffected vegetative ponds (Sites and Vitheepradit 2011). Obviously, the richness of aquatic and semi-aquatic true bugs recovered during this research was much lower than previous studies, possibly due to the scale of the study area. In general, spatial scale is positively correlated with species richness of aquatic insects (Resh and Rosenberg 1989, Gledhill et al. 2008, Heino 2009, Hassall et al. 2011, Bacca et al. 2020). A larger study area results in more species collected. The study area of this research is less than 1% of the total land of the country, whereas previous studies focused on a distinctly larger area that included northern, western and southern regions (Vitheepradit 2008, Sites and Vitheepradit 2011). Therefore, the species richness collected during this research was in the expected range, representing 9% of all known freshwater aquatic and semi-aquatic species known from Thailand (Vitheepradit, unpublished data).

A wide range of biotic and abiotic factors influence both richness and diversity of aquatic insects, including water quality, the presence of fish, the stability of the water level, the canopy cover and plant richness (Voelz and McArthur 2000, Keinath et al. 2023). Plant diversity plays an important role in shaping the species richness and assemblages of aquatic and semi-aquatic insects in both lotic and lentic systems (Jeffries 2005, Hassall et al. 2011, Nakanishi et al. 2013, Briggs et al. 2019). Although aquatic and semi-aquatic true bugs are predaceous, many taxa of Nepomorpha are associated with the presence of plants (Andersen and Weir 2004, Chen et al. 2021). Members of Nepidae and Belostomatidae are commonly found in water amongst aquatic vegetation waiting to capture prey (Andersen and Weir 2004). Species of Notonectidae have been reported to lay eggs on submerged parts of aquatic plants (Chen et al. 2021). In lotic systems, species diversity and richness of aquatic and semi-aquatic true bugs were higher at collecting sites with abundant riparian and vegetation along the margins than those with an insignificant number of plants at margins (Dias-Silva et al. 2010, Attawanno and Vitheepradit 2022). Whereas, results of several research indicated there was no significant relationship between Gerromorpha and the presence of marginal vegetation in habitats (Dias-Silva et al. 2020). Therefore, the relationship of Nepomorpha and Gerromorpha with marginal vegetation are in need for further investigation. Nevertheless, the existence of plants at margins of natural ponds in this study created more suitable habitats for members of Nepomorpha to occupy, which subsequently promotes higher richness and abundance of aquatic and semi-aquatic true bugs. Therefore, the existence of plants at naturally-margined ponds in this study created more suitable habitats for members of Nepomorpha to occupy, which subsequently promotes higher richness and abundance of aquatic and semi-aquatic true bugs. Urbanisation is recognised as having a major impact on insect diversity (Ye et al. 2013, Adams et al. 2020, Fenoglio et al. 2021). Habitat loss and fragmentation have been determined to highly affect insect communities in urban areas (Adams et al. 2020, Gaona et al. 2021, Vaz et al. 2023). Unlike plant diversity, insect diversity is not easily managed by humans due to the complex associations with abiotic and biotic factors required by insects (Adams et al. 2020). Nevertheless, green zones or parks are vital to maintain diversity of insects in urban areas (Peng et al. 2020). The landscape within and surrounding Bangkok has been rapidly altered over the past several decades (Mekvichai et al. 1990, Thaweepworadej 2021). Specifically, the lotic systems in Bangkok have been changed to serve human activities, including transportation, irrigation and drainage (Mekvichai et al. 1990, Thaweepworadej 2021). Therefore, lentic systems, found throughout parks in the green zone in Bangkok, have become the last available habitat for aquatic insects. Ponds in parks managed by the BMA provide suitable and stable habitats for aquatic insects that can tolerate stresses from human activities. That may possibly explain why all the taxa collected during this research are common species that are widely distributed throughout the country (Vitheepradit 2000, Cheng et al. 2001, Nieser 2004, Vitheepradit 2008, Polhemus and Polhemus 2013, Cook et al. 2020).

The results of this research provide valuable knowledge in both taxonomic and ecological aspects. Taxonomically, this research presents the first species-level list of aquatic and semi-aquatic true bugs inhabiting the green zone of Bangkok. Ecologically, this research has shown that the presence of vegetation at the pond margin can promote higher richness and abundance of aquatic insects, especially aquatic and semi-aquatic true bugs that potentially can be used as biological control of mosquito larvae (Shaalan and Canyon 2009, Brahma et al. 2014). The results of this research also contribute fundamental information for future research in biodiversity and better park management within Bangkok. This research is an important initial step for understanding biodiversity of aquatic insects within the green zones of Bangkok. The relationship of aquatic insect diversity and various impacts from urbanisation (e.g. light intensity, barrier of construction, human density) remain unexplored. Additional studies on diversity and richness of aquatic insects within ponds under different management regimes may reveal important management techniques. Additionally, the presence of floating plants in the ponds may maximise diversity of aquatic insects in urban ponds. Clearly, more research is needed to gain a better understanding of proper management to improve and conserve biodiversity in Bangkok.

## Acknowledgments

We are grateful to Dr. La-au Nakthong (Kasetsart University) Dr. Prabseuk Sritipsak (Kasetsart University) and Dr. Sajeemat Attawanno (Thailand Institute of Nuclear Technology) and students in Systematic and Ecological Entomology Laboratory at Kasetsart University (SEEK Lab), including Ms. Areerat Khenmee, Mr. Pattarawich Dawwrueng and Ms. Thitinat Khongkhieo for their assistance in the field. We are thankful to the Bangkok Metropolitan Administration (BMA) for allowing the research in the topic “Diversity of aquatic insects in the recreational parks in Bangkok” in their recreational parks. This research was approved by the Institutional Animal Care and Use Committee, Faculty of Agriculture, Kasetsart University, Thailand under Project number ACKU66-AGR-004 This research was funded by the Kasetsart University Research and Development Institute, KURDI (Grant No. FF (KU) 51.68).

## Figures and Tables

**Table 1.** Name, locality and margin type of ponds surveyed during this research.

**Pond Number**	**District**	**Recreation Park**	**N**	**E**	**Margin type**
L1	Nong Chok	Rat Phirom Public Park	13.8079	100.8822	Natural
L2	Min Buri	Phraya Phirom Park	13.8279	100.7631	Natural
L3	Lat Krabang	Wanapirom Romklao Park	13.7553	100.7457	Natural
L4	Bang Bon	His Majesty The King Chaloem Phra Kiat Public Park, Bang Bon	13.6492	100.3966	Natural
L5	Thawi Watthana	Thawi Wanarom Park	13.7441	100.3523	Natural
L6	Bangkok Noi	Sirinthra Phrueksa Phan Park	13.7467	100.4668	Natural
L7	Bangkok Noi	His Majesty King Bhumibol Adulyadej 80th birthday anniversary Park	13.7719	100.4683	Natural
L8	Prawet	Chaloem Phra Kiat Mahat Thai Park	13.6767	100.6779	Natural
L9	Khlong Toei	Benchakitti Forest Park	13.7285	100.5537	Natural
L10	Bang Kho Laem	Public Park in Commemoration of H.M. the King's 6th Cycle Birthday	13.6854	100.5182	Natural
L11	Bang Khen	Ramindra Sport Park	13.8780	100.6205	Natural
L12	Chatuchak	Wachirabenchatat Park	13.8131	100.5547	Natural
L13	Phra Nakhon	Rommaninat Park	13.7494	100.5027	Artificial
L14	Phra Nakhon	Saranrom Park	13.7483	100.4949	Artificial
L15	Chatuchak	Queen Sirikit Park	13.8076	100.5507	Artificial
L16	Chatuchak	Chatuchak Park	13.8076	100.5555	Artificial
L17	Khlong Toei	Benchasiri Park	13.7316	100.5679	Artificial
L18	Khlong Toei	Benchakiti Park	13.7296	100.5587	Artificial
L19	Phra Nakhon	Santiphap Park	13.7630	100.5413	Artificial
L20	Bang Khae	Bang Khae Phirom Park	13.6863	100.3855	Natural
L21	Thung Khru	Thonburirom Park	13.6521	100.4916	Artificial
L22	Bueng Kum	Seri Thai Park	13.7871	100.6743	Artificial
L23	Lat Krabang	Her Majesty the Queen's 60th Birthday Park	13.7631	100.7323	Natural
L24	Lat Krabang	Phra Nakhon Park	13.7210	100.7812	Natural
L25	Prawet	Wanadharm Park	13.6839	100.6567	Natural
L26	Prawet	Bueng Nong Bon Park	13.6968	100.6595	Natural
L27	Nong Chok	Nong Chok Park	13.8545	100.8595	Artificial
L28	Khlong Sam Wa	Wari Phirom Park	13.8489	100.7766	Natural

**Table 2.** Species and number of specimens collected during this study. The first number refers to a number of specimens collected in a margin type and the second number refers to number of ponds from which each species was collected.

**Infraorder**	**Family**	**Genus**	**Species**	**Margin type**	
				**Natural**	**Artificial**
Gerromorpha	Gerridae	*Aquarius*	*A.adelaidis* Dohrn, 1860	64/18	2/10
		*Limnogonus*	*L.fossarum* (Fabricius, 1775)	239/18	73/10
		*Naboandelus*	*N.signatus* Distant, 1910	0	12/10
		*Rhagadotarsus*	*R.kraepelini* Breddin, 1905	635/18	59/10
	Hebridae	*Hebrus*	*Hebrus* sp. 1	1/18	0
	Hydrometridae	*Hydrometra*	*H.cracens* Polhemus & Polhemus, 1995	15/18	4/10
	Mesoveliidae	*Mesovelia*	*M.horvathi* Lundblad, 1933	241/18	179/10
			*M.vittigera* Horváth, 1895	108/18	90/10
	Veliidae	*Microvelia*	*M.douglasi* Scott, 1874	34/18	152/10
Nepomorpha	Belostomatidae	*Diplonychus*	*D.rusticus* Fabricius, 1871	67/18	24/10
	Helotrephidae	*Tiphotrephes*	*T.indicus* (Distant, 1910)	34/18	3/10
	Micronectidae	*Micronecta*	*M.scutellaris* (Stål, 1858)	362/18	29/10
			*M.grisea* (Fieber, 1844)	709/18	93/10
	Naucoridae	*Thurselinus*	*T.scutellaris* (Stål, 1860)	49/18	14/10
	Nepidae	*Ranatra*	*R.longipes* Stål, 1861	41/18	0
	Notonectidae	*Anisops*	*A.breddini* Kirkaldy, 1901	729/18	28/10
		*Nychia*	*N.sappho* Kirkaldy, 1901	394/18	16/10
	Pleidae	*Paraplea*	*P.frontalis* (Fieber, 1844)	165/18	7/10
			*P.lateromaculata* Cook, 2020	26/18	0
			*P.liturata* (Fieber, 1844)	22/18	0

**Table 3.** Student’s t-test table showing the difference of abundance of Gerromorpha, Nepomopha and All taxa.

**Independent Samples test**										
		**F**	**Sig.**	**t**	**df**	**Sig.(2-tailed)**	**Mean Difference**	**Std.Error Difference**	**95% Confidence Interval of the Difference**	
									**Lower**	**Upper**
Gerromorpha	Equal variances assumed Equal variances not assumed	2.411	0.133	0.725	26	0.475	19.344	26.666	-35.469	74.158
				0.871	25.769	0.392	19.344	22.210	-26.328	65.017
Nepomorpha	Equal variances assumed Equal variances not assumed	5.583	0.026	2.142	26	0.042	117.178	54.700	4.739	229.616
				2.841	18.943	0.010*	117.178	41.247	30.828	203.527
All taxa	Equal variances assumed Equal variances not assumed	6.487	0.017	2.223	26	0.035	136.522	61.424	10.263	262.781
				2.913	20.081	.009*	136.522	46.873	38.772	234.273
